# Reduced levels of two modifiers of epigenetic gene silencing, Dnmt3a and Trim28, cause increased phenotypic noise

**DOI:** 10.1186/gb-2010-11-11-r111

**Published:** 2010-11-19

**Authors:** Nadia C Whitelaw, Suyinn Chong, Daniel K Morgan, Colm Nestor, Timothy J Bruxner, Alyson Ashe, Eleanore Lambley, Richard Meehan, Emma Whitelaw

**Affiliations:** 1 Genetics and Population Health, Queensland Institute of Medical Research, 300 Herston Road, Brisbane, Queensland 4006, Australia; 2 School of Medicine, University of Queensland, 288 Herston Road, Brisbane, Queensland 4001, Australia; 3 MRC Human Genetics Unit, Institute of Genetics and Molecular Medicine, Crewe Road, Edinburgh EH4 2XU, UK; 4 Breakthrough Research Unit, University of Edinburgh, Crewe Road, Edinburgh EH4 2XU, UK

## Abstract

**Background:**

Inbred individuals reared in controlled environments display considerable variance in many complex traits but the underlying cause of this intangible variation has been an enigma. Here we show that two modifiers of epigenetic gene silencing play a critical role in the process.

**Results:**

Inbred mice heterozygous for a null mutation in *DNA methyltransferase 3a *(*Dnmt3a*) or *tripartite motif protein 28 *(*Trim28*) show greater coefficients of variance in body weight than their wild-type littermates. *Trim28 *mutants additionally develop metabolic syndrome and abnormal behavior with incomplete penetrance. Genome-wide gene expression analyses identified 284 significantly dysregulated genes in *Trim28 *heterozygote mutants compared to wild-type mice, with *Mas1*, which encodes a G-protein coupled receptor implicated in lipid metabolism, showing the greatest average change in expression (7.8-fold higher in mutants). This gene also showed highly variable expression between mutant individuals.

**Conclusions:**

These studies provide a molecular explanation of developmental noise in whole organisms and suggest that faithful epigenetic control of transcription is central to suppressing deleterious levels of phenotypic variation. These findings have broad implications for understanding the mechanisms underlying sporadic and complex disease in humans.

## Background

Experiments designed to analyze the significance of genes and environment on quantitative traits using laboratory rats and mice have found that 70 to 80% of all variation is of unknown origin [1]. Gartner [2] carried out experiments over a period of 20 years to analyze the significance of different components of random variability in quantitative traits. Reduction of genetic variability, by using inbred strains, and reduction of environmental variability, by standardized husbandry, did not significantly reduce the range of random phenotypic variability. Similarly, moving the animals into the wild to increase environmental variability did not increase random phenotypic variability, hence the term 'intangible variance' [1]. For example, only 20 to 30% of the range of the body weights of inbred mice was estimated to be the result of postnatal environment, with the remaining 70 to 80%, which Gartner termed 'the third component', being of unknown origin. These and other studies suggested that this phenotypic variation, also known as 'developmental noise' [3], is determined early in ontogeny [4,5].

Comparisons of classic quantitative traits, such as body weight and behavior, across mouse strains have been hampered by the difficulty of controlling for maternal effects. In the experiments described here, such effects have been ruled out by comparing mutant with wild-type littermates, raised in the same cage by the same dam. The studies have been carried out using mice heterozygous for known modifiers of epigenetic reprogramming, one of which (*Trim28^MommeD9/+^*) emerged from a dominant screen for modifiers of epigenetic reprogramming. In this screen *N*-ethyl-*N*-nitrosourea (ENU) mutagenesis was carried out on inbred FVB/NJ mice carrying a variegating GFP transgene expressed in red blood cells [6]. The percentage of cells expressing the transgene is sensitive to the dosage of epigenetic modifiers. The screen has identified both known (*Dnmt1*, *Smarca5*, *Hdac1*, *Baz1b*) and novel (*SmcHD1*) genes [7-9] and has provided us with mouse models (*MommeD*s) to study the role of epigenetic reprogramming in whole organisms and populations.

Mice with reduced levels of DNA methyltransferases [10] and other modifiers of epigenetic reprogramming (for example, Suv39 h, Hdac1, Smarca5, Mel18) are viable, reproduce and are superficially phenotypically normal [11-13]. We were keen to discover subtle phenotypic abnormalities in *MommeD *mice and found that cohorts heterozygous for some modifiers of epigenetic gene silencing display greater phenotypic noise.

## Results

**Figure 1:**
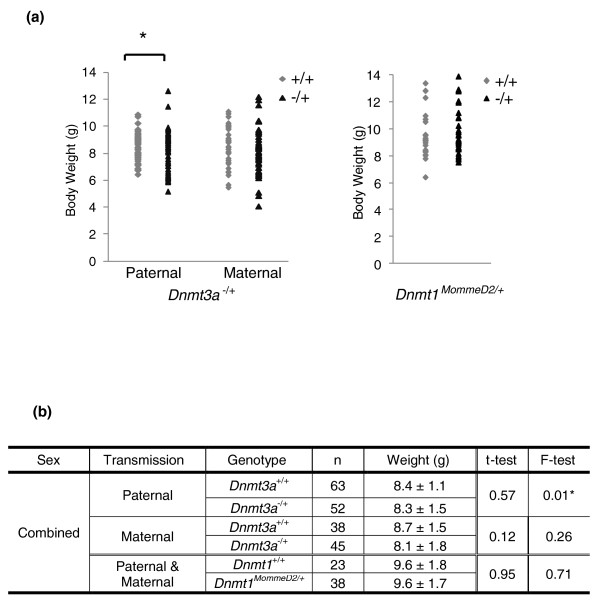
**Variance in weights of mice haploinsufficient for Dnmt3a**. **(a)** Mice from paternal and maternal transmission of the *Dnmt3a*^- ^null allele and the *Dmnt1^MommeD2 ^*allele were weighed and genotyped at 3 weeks of age (weaning). The data presented in these graphs are tabulated below. **(b) **There is significantly more variation in the weights of *Dnmt3a^-/+ ^*mice following paternal transmission of the mutant allele (F test, *P *= 0.01). *Dnmt3a*^- ^data were collected from wild-type and heterozygous mutant littermates from a wild-type x heterozygous cross. *Dmnt1^MommeD2 ^*data were collected from wild-type x heterozygous crosses (equal contributions from reciprocal crosses) and heterozygous intercrosses.

In the experiments described here the colonies were maintained by backcrossing to the inbred congenic strain, in some cases C57BL/6 and in other cases FVB/NJ, and offspring were weighed at weaning. A knockout allele of *Dnmt3a*, *Dnmt3a*^-^, a gift from En Li, was backcrossed for 11 generations to C57BL/6 and subsequently maintained in that background. Homozygosity for this allele (in the original mixed genetic background) has been shown to result in runting and death in the early postnatal period [14], but no phenotypic abnormalities were reported for heterozygous individuals. Here we show that in the inbred C57BL/6 background, haploinsufficiency for Dnmt3a was associated with a trend towards reduced body weight, a larger standard deviation from the mean and a significantly increased coefficient of variance compared to wild-type littermates (Figure 1). This effect appears to be more marked following paternal inheritance of the mutant allele but this could be the result of the larger dataset (Figure 1). In all cases the ratio of males to females was similar (data not shown). This result argues that reduction in the level of DNA methyltransferase 3a results in increased developmental noise.

We were keen to discover whether similar effects would be seen with other proteins involved in epigenetic reprogramming. We have previously reported that *MommeD2 *mice carry a mutation in the *Dnmt1 *gene that destabilizes the protein and heterozygotes are haploinsufficient for Dnmt1 [7]. This mouse strain was produced and maintained on the FVB/NJ background. In *Dnmt1^MommeD2/+^* mice there was no difference in the mean, the range, or the coefficient of variance of body weight at weaning (Figure 1). Similarly, we have published previously that haploinsufficiency for Snf2 h (the protein disrupted in *Smarca5^MommeD4/+ ^*mice) resulted in smaller mean body weight but with no obvious increase in the coefficient of variance [7] and that haploinsufficiency for Baz1b (the protein disrupted in *Baz1b^MommeD10^* mice) resulted in no change to the mean body weight, nor the coefficient of variance [9].

**Figure 2:**
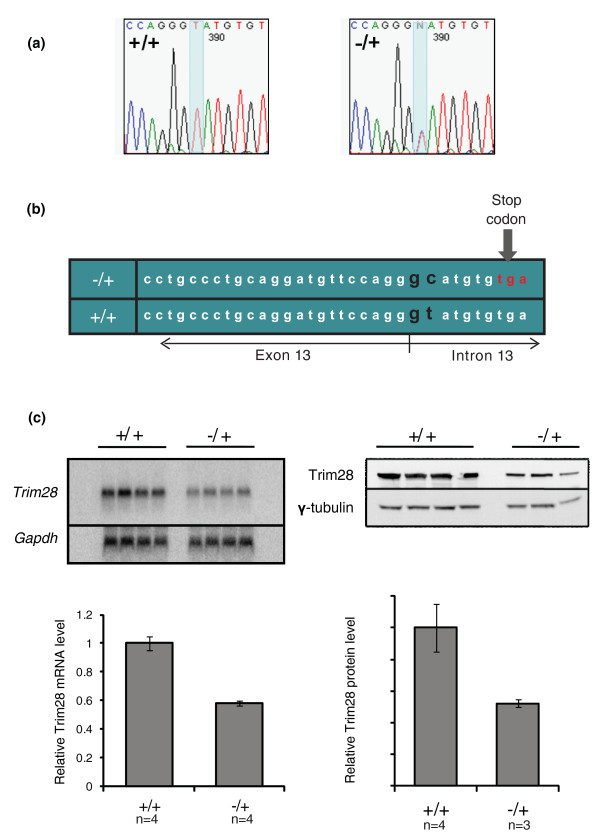
**Haploinsufficiency for *Trim28 *caused by a splice site mutation**. **(a)** Sequence chromatograms show that *MommeD9^-/+ ^*mice have a T to C mutation 2 bp into intron 13 of *Trim28*. **(b) **The mutation is expected to prevent splicing of intron 13 causing an in-frame premature stop codon. The splice acceptor site is shown in black. (**c**) Northern and western analysis of *Trim28 *mRNA and protein show that *MommeD9^-/+ ^*mice have a reduced dosage of Trim28. Error bars indicate + SEM.

Mice heterozygous for the *MommeD9 *mutation are viable and have a decrease in the percentage of red blood cells expressing GFP, that is, the gene is an enhancer of variegation [9]. Homozygous individuals die prior to midgestation and linkage analysis revealed that the mutation lies on chromosome 7 (mm8) [9]. We have now reduced the interval to a 3.4-Mb region (between rs31712695 and rs32435505) containing 52 genes (Additional file 1). The best candidate gene was *Trim28 *(also known as *Kap1*), a gene that codes for a bromodomain-containing protein. The human homolog has been shown to form a complex with heterochromatin protein 1 (HP1), histone deacetylase 1 (HDAC1) and the histone methyltransferase SETDB1 [15,16]. Sequencing of exons and intron-exon boundaries revealed a T to C point mutation 2 bp into intron 13 of *Trim28 *in mutant individuals (Figure 2a). This has been verified in over 100 mice. The mutation is predicted to prevent correct splicing and introduce a premature stop codon (Figure 2b). Northern and western analysis revealed half the level of *Trim28 *mRNA and protein in the heterozygous mutants (Figure 2c), presumably the result of nonsense-mediated mRNA decay of the mutant transcript. No abnormally sized mutant transcripts were observed. Assuming an ENU-induced mutation rate of 1 in 1.5 Mb, the probability of a second mutation in the coding region of this interval is extremely low (*P *= 0.0006 [17]). Based on these findings, in combination with the fact that homozygous mutant embryos [9] die at the same stage as that reported for the *Trim28 *knockout allele [18], we designated the mutant allele *Trim28^MommeD9^*.

**Figure 3:**
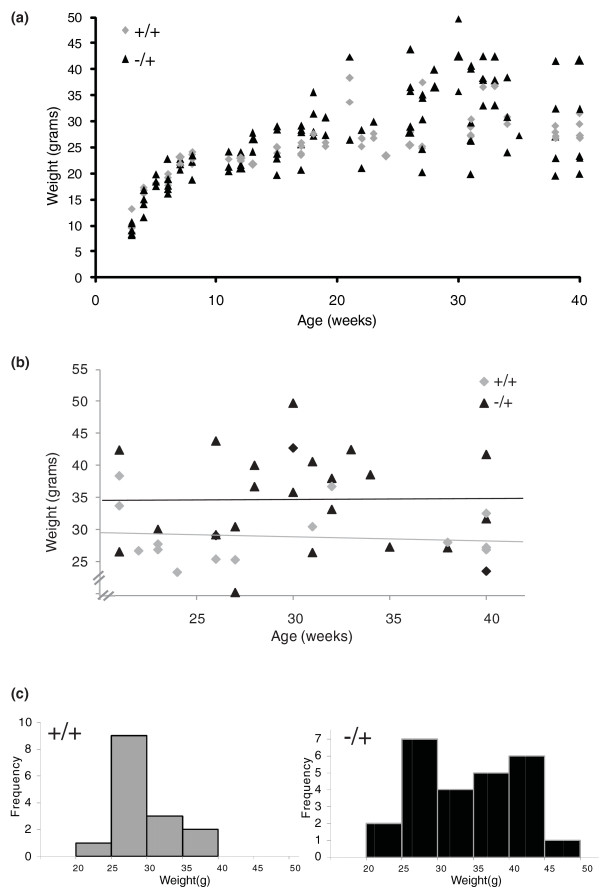
**Increased variation in body weight in *Trim28^MommeD9/+ ^*mice**. **(a)** Twenty *Trim28^+/+ ^*mice and 33 *Trim28^MommeD9/+ ^*mice (all female) were weighed between 3 and 40 weeks of age. The data are the sum of 170 data points representing 103 *Trim28^MommeD9/+ ^*and 67 *Trim28^+/+ ^*body weight measurements. *Trim28^MommeD9/+ ^*mice appear to have a greater variation in weight as they age. **(b) **There is no correlation between age and weight between 20 and 40 weeks of age in 15 *Trim28^+/+ ^*mice and 25 *Trim28^MommeD9/+ ^*mice; however, *Trim28^MommeD9/+ ^*mice are heavier on average (*P *= 0.008). (**c) ***Trim28^MommeD9/+ ^*mice have a significant increase in weight variation between the ages of 20 and 40 weeks (*P *= 0.005).

**Figure 4:**
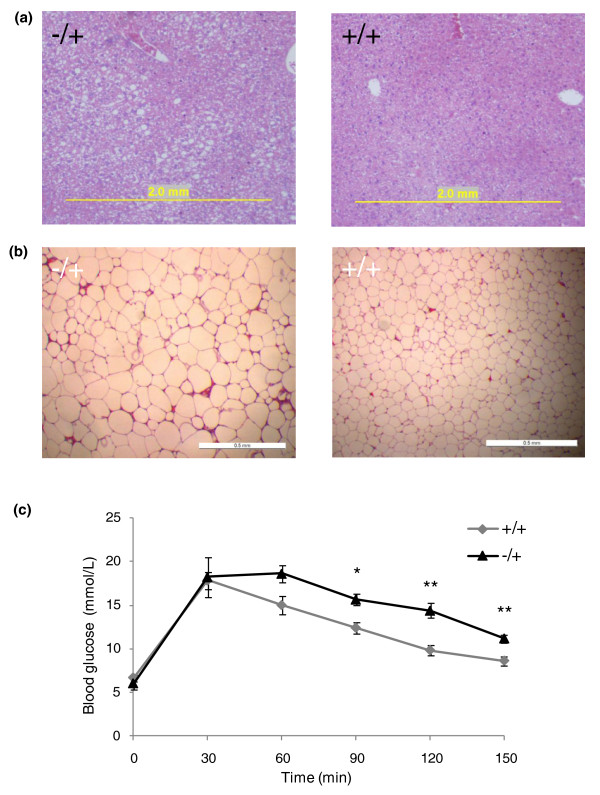
**Symptoms of metabolic syndrome in obese *Trim28^MommeD9/+ ^*mice**. **(a)** Liver tissue was dissected from an obese *Trim28^MommeD9/+ ^*mouse and a wild-type littermate. Tissues were sectioned and stained with H&E. **(b) **Inguinal fat pads were dissected from an obese *Trim28^MommeD9/+ ^*mouse and a wild-type littermate. Tissues were sectioned and stained with H&E. In both cases the data shown are representative of sections taken from at least five different *Trim28^MommeD9/+ ^*mutants and five different *Trim28^+/+ ^*individuals. **(c) **Four obese *Trim28^MommeD9/+ ^*mice and six *Trim28^+/+ ^*littermates were fasted for 15 hours and a blood glucose measurement was taken at t = 0. Mice were injected with 2 g/kg of a 20% glucose solution and blood glucose measurements were taken every 30 minutes for 150 minutes with a blood glucose monitor (Accu-Chek). *P < 0.05, **P < 0.005 (Students *t*-test).

As they age, some but not all female *Trim28^MommeD9/+ ^*mice became obese (Figure 3a). The body weights of female *Trim28^MommeD9/+ ^*mice and wild-type littermates between the ages of 3 and 40 weeks were measured. In this original data set, some individuals were weighed at more than one time point. When a single observation per mouse was randomly selected between the ages of 20 and 40 weeks (Figure 3b,c), the mean body weight of *Trim28^MommeD9/+ ^*females (34.2 ± 7.6 grams, n = 25) was greater than that of wild-type female littermates (28.9 ± 4.3 grams, n = 15; independent samples *t*-test with unequal variances, *P *= 0.008) and the coefficient of variance was also greater in *Trim28^MommeD9/+ ^*females (Levene's test, *P *= 0.005). Obesity was associated with liver steatosis, adipocyte hypertrophy and impaired glucose tolerance (Figure 4). Taken together, these results show that mice with a half dosage of *Trim28 *are predisposed to metabolic syndrome [19]. Some isogenic littermates do not display this phenotype, demonstrating a significant degree of stochasticity in the development of metabolic syndrome in *Trim28 *heterozygous mutants.

**Figure 5:**
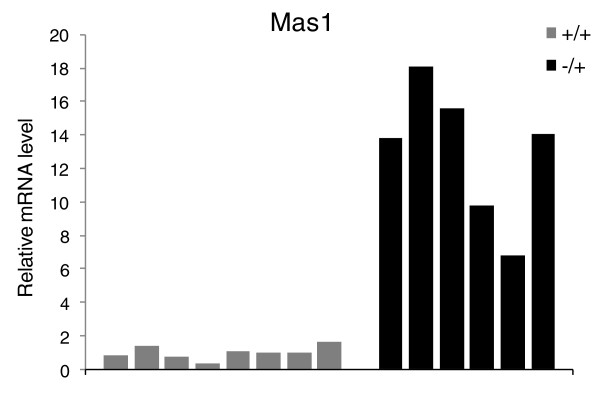
**Variable expression of *Mas1 *in *Trim28^MommeD9/+ ^*mice**. Expression levels of the *Mas1 *gene were validated by quantitative PCR on cDNA from additional *Trim28^MommeD9/+ ^*(n = 6) and wild-type individuals (n = 8). Levels were normalized to *Gapdh*.

In an attempt to identify the genes that respond directly to reduced levels of Trim28, we carried out a genome-wide expression analysis (Illumina MouseRef-8 v2.0 Expression BeadChip) using RNA from livers of 4-week-old male *Trim28^MommeD9/+ ^*individuals (n = 4) and their wild-type male littermates (n = 4). At 4 weeks of age heterozygous mutants are not heavier than their wild-type littermates (Figure 3a) and their livers show no obvious pathology (data not shown). This time point was chosen in the hope of detecting initiating events. There were 59 genes significantly up-regulated in *Trim28^MommeD9/+ ^*individuals and 225 genes were significantly down-regulated (Additional file 2). The proto-oncogene *Mas1 *was expressed 7.8-fold higher in *Trim28^MommeD9/+ ^*individuals and was the most significant change. Quantitative PCR validation in additional sex and age-matched samples revealed that the expression level of *Mas1 *is highly variable across mutant mice (Figure 5; F test, *P *< 0.005). Mas1 is a G-protein coupled receptor recently identified as playing a central role in lipid metabolism and metabolic syndrome [20]. Ingenuity Pathway Analysis of dysregulated genes revealed that the two top canonical pathways were 'LPS/IL-1 mediated inhibition of RXR function' and 'Glycine, serine and threonine metabolism' while the two top gene networks (scores of 46 and 42) functioned in 'Tissue morphology, cell death, infection mechanism' and 'Hepatic system disease, liver cholestasis, lipid metabolism'. These results suggest that haploinsufficiency for *Trim28 *leads to a gene dysregulation signature in the liver, possibly via Mas1, that may be predictive of developing metabolic disease later in life. We were interested in testing whether epigenetic regulatory mechanisms such as CpG methylation and histone methylation are important features in the control of gene expression by Trim28. Promoter classification analysis using previously published genome-wide methylation and histone mapping data [21] was performed on all genes classed as up-and down-regulated by the GenomeStudio (Illumina) analysis. The promoters of genes down-regulated in *Trim28^MommeD9/+ ^*individuals had a higher CpG density and a higher histone H3 lysine 4 trimethylation density (Additional file 3), suggesting that much of the gene dysregulation in mutant mice is targeted to a subset of genes with characteristic epigenetic features. These promoter regions may harbor epimutations that cause the mutant phenotypes in *Trim28^MommeD9/+ ^*mice.

**Figure 6:**
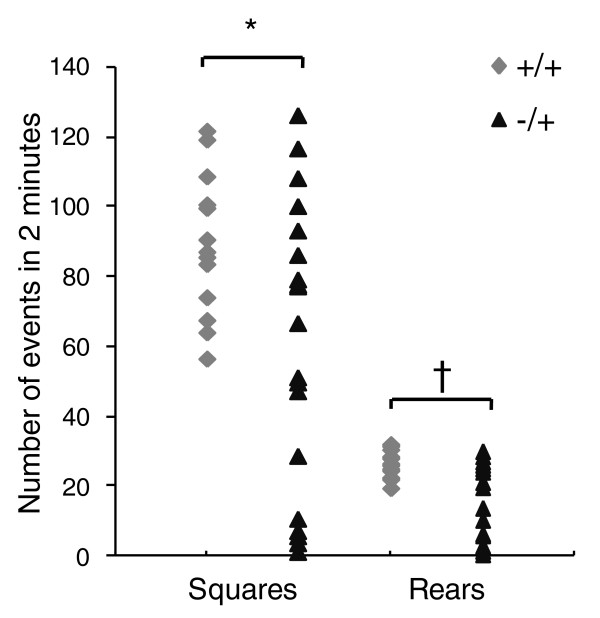
**Abnormal exploratory behavior in *Trim28^MommeD9/+ ^*mice**. The behavior of 19 *Trim28^MommeD9/+ ^*mice and 14 *Trim28^+/+ ^*mice was tested in an open field (40 cm × 40 cm). Mice were scored for the number of 10-cm^2 ^squares entered (Squares) and the number of times they reared on their hind legs (Rears) in a 2-minute period. *P < 0.05 (*t*-test and F test), ^†^*P *< 0.0005 (*t*-test and F test).

A recent study of a *Trim28 *conditional knockout in the forebrain reported heightened anxiety and stress-induced behavior in mutant animals [22]. We tested the *Trim28^MommeD9/+ ^*adult mice in an open field test and found that some, but not all, individuals displayed reduced exploratory behavior as measured by both squares entered and the frequency of rearing on their hind legs (Figure 6). Again, the coefficient of variance in the mice haploinsufficient for *Trim28 *was significantly greater than that found in their wild-type littermates. *Trim28^MommeD9/+ ^*individuals also showed an increased frequency of defecation (5 of 19 mutants compared to 0 of 14 wild types during the test period), consistent with increased anxiety. There was no correlation between the mice that behaved abnormally in the behavioral test and body weight (Additional file 4).

## Discussion

Transcriptional noise at the cellular level has been documented in single cell organisms [23,24]. Gordon and colleagues [25] have shown, using single cell observation of the bistable *lac *operon in *Escherichia coli*, that reduction in the levels of proteins regulating transcription can result in heritable aberrant behavior in genetically identical cells. Intrinsic variability in expression state at a number of genes in yeast has been shown to be associated with changes in the epigenetic state of their promoters [26-28]. The manifestation of this transcriptional noise at the level of multicellular organisms or populations is rarely considered. Interestingly, Raj and colleagues [29] have recently shown that increased transcriptional noise can lead to intestinal cell fate changes in *Caenorhabditis elegans *and that chromatin proteins may be involved. Our data are consistent with this finding. Here we have shown that reduced levels of two proteins involved in transcriptional gene silencing, Dnmt3a and Trim28, cause increased phenotypic variance in inbred littermates.

While developmental flexibility with respect to cell fate is necessary for complex organisms to produce multiple cell types, unfettered transcriptional noise appears to be detrimental. Not all inbred colonies haploinsufficient for epigenetic modifiers display changes in body weight (for example, Baz1b [9], Dnmt1) but more extensive phenotypic analysis using a broader range of measurements may reveal other traits with increased variation. Perhaps transcriptional noise at critical stages in early development results in increased variance in cell fate decisions among mutant offspring leading to changes in the proportions of different tissue types in the adult. While it is theoretically possible that reduced levels of epigenetic modifier proteins lead to increased genetic changes, we see no evidence of this using comparative genomic hybridization arrays (data not shown). Our data suggest that disrupting the epigenome can change gene regulatory networks and that this results in increased phenotypic variation.

## Conclusions

The capacity of an organism to ensure the production of a standard phenotype in spite of environmental disturbances is called canalization [30]. Our studies show that modifiers of epigenetic gene silencing are fundamental to this process and suggest that their levels have been fine-tuned by evolutionary pressures to allow cells to acquire different patterns of gene expression during differentiation, but at the same time to lock-in the transcriptional profile of differentiated cell types. Numerous studies in vertebrates and invertebrates using isogenic individuals raised in controlled environments show considerable variance for many phenotypic traits, for example, body weight and bristle number. This is the first report of any mechanism that can change the degree of variance at the level of the whole organism in mammals. Our findings have broad implications for the mechanisms underlying phenotype and disease in all multicellular organisms.

## Materials and methods

### Mouse strains and genotyping

Wild-type inbred C57BL/6J mice were purchased from ARC Perth (Perth, WA, Australia). Procedures were approved by the Animal Ethics Committee of the Queensland Institute of Medical Research. The ENU screen was carried out in an FVB/NJ inbred line that carry a GFP transgene, as described previously [6]. *Dnmt1^MommeD2 ^*mice and *Trim28^MommeD9 ^*mice were maintained in this background unless stated otherwise. *Dnmt1^MommeD2 ^*mice and *Trim28^MommeD9 ^*mice were classed as heterozygous or wild-type by fluorescence-activated cell sorting (FACS) analysis of GFP expression as described previously [7,9]. The *Dmnt3a^- ^*knockout allele was maintained on a C57BL/6 background and detected by PCR primers specific for the *neo *cassette, as described at the Jackson Laboratory website [31].

### Linkage analysis

FVB/NJ *MommeD9 *heterozygotes, homozygous for the GFP transgene, were backcrossed twice to C57BL/6 and phenotyped for GFP expression by flow cytometry, as previously described [9]. DNA from tail tips was used to perform a genome-wide linkage scan, which identified the linked interval on chromosome 7 [9]. We have reduced the linked interval from that reported by using additional SNP markers. Fine mapping using microsatellite and SNP markers polymorphic between FVB/NJ and C57BL/6 was carried out on 127 wild types and 103 heterozygotes to define the linked interval. Estimating the probability of ENU-induced coding mutations was performed using formulas accessible on the 'enuMutRat on zeon' website [32].

### RNA and cDNA analysis

Poly(A)^+ ^RNA was purified from the livers of 4-week-old male *Trim28^MommeD9/+ ^*mice and *Trim28^+/+ ^*littermates. RNA was separated on a 1% denaturing agarose gel, transferred and hybridized with a fragment encompassing *Trim28 *exons 11 and 12 using PCR primers (Additional file 2). cDNA was prepared from total RNA from the livers of 4-week-old *Trim28^MommeD9/+ ^*mice and *Trim28^+/+ ^*littermates using random priming and the Superscript^®^III system (Invitrogen, Carlsbad, CA, USA). Quantitative RT-PCR reactions were prepared using SYBR^® ^Green PCR Master Mix (Applied Biosystems, Carlsbad, CA, USA). PCRs were run on standard programs using a Rotor-Gene 3000 (Corbett/Qiagen, Valencia, CA, USA). *Mas1 *mRNA was amplified with primers: 5′-AAGCCTCTAGCCCTCTGTCC-3′ (forward) and 5′-GGTCCATGAGGAGTTCTTGA-3′ (reverse).

### Protein analysis

Nuclear extracts were prepared from the spleens of 4-week-old *MommeD9 *mice. Approximately 5 μg of proteins were separated by SDS-PAGE on a 4 to 12% Bis-tris polyacrylamide gel (Invitrogen) and were analyzed with a monoclonal antibody to Trim28 (MAB3662, Millipore, Billerica, MA, USA).

### Expression arrays

For Illumina BeadArray analysis, total liver RNA from *Trim28^MommeD9/+ ^*mice (n = 4) and *Trim28^+/+ ^*mice (n = 4) was assessed for integrity using the Agilent Bioanalyzer 2100, and RNA integrity (RIN) scores above 8 were present in all samples. RNA was amplified using the Illumina TotalPrep RNA Amplification kit (Ambion, Carlsbad, CA, USA). Amplified cRNA was assessed for quantity and quality also using the Agilent Bioanalyzer 2100. RNA was hybridized to MouseRef-8 v2.0 Expression BeadChip (Illumina, Carlsbad, CA, USA) according to the manufacturer's instructions. Technical replicates were performed for all samples. BeadChip arrays were scanned with Illumina BeadStation Scanner and data values with detection scores were compiled using BeadStudio (Illumina). The gene expression data were analyzed by the GenomeStudio Gene Expression Module (Illumina). Genes with significantly different expression (difference score > 16) were analyzed using Ingenuity Pathway Analysis. The expression data have been deposited in NCBI's Gene Expression Omnibus (GEO), and is accessible through GEO Series accession number [GEO:GSE23512] [33].

### Behavioral testing

*Trim28^MommeD9/+ ^*mice and *Trim28^+/+ ^*littermates between the ages of 5 and 11 months were placed into a 40 cm × 40 cm box with a grid dividing it into 16 squares (10 × 10 cm). Mice were placed in the open field and scored for the number of squares entered, and number of times the mouse reared up on its hind legs over a 2-minute period. Data were collected by two independent investigators, one of whom was blind to genotype. The data were the average of the two scores and scores were 90% concordant.

## Abbreviations

ENU: *N*-ethyl-*N*-nitrosourea; GEO: Gene Expression Omnibus; GFP: green fluorescent protein; H&E: haematoxylin and eosin; SNP: single nucleotide polymorphism.

## Authors' contributions

NCW, SC, DKM, CN, TJB, AA, EL and RM carried out the experiments and helped to draft the manuscript. EW conceived of the study, participated in its design and coordination and helped to draft the manuscript. All authors read and approved the final manuscript.

## Supplementary Material

Additional file 1 **Table S1**. List of genes in the *MommeD9 *linked interval. Click here for file

Additional file 2 **Table S2**. Gene expression analysis of *Trim28^MommeD9/+ ^*mice. Click here for file

Additional file 3 **Figure S1**. Promoter characteristics of aberrantly expressed genes in *Trim28^MommeD9/+ ^*mice. Genome-wide expression analysis (Illumina MouseRef-8 v2.0 Expression BeadChip) was performed using RNA from the livers of 4-week-old male *Trim28^MommeD9/+ ^*individuals (n = 4) and their wild-type littermates (n = 4). Promoter classification analysis was performed on genes classed as upregulated (n = 59) and downregulated (n = 225) by the GenomeStudio Gene Expression Module (Illumina). (**a**) Promoters were classified as low (LCP), intermediate (ICP) or high (HCP) CpG density. (**b**) Promoters were classified as having histone 3 lysine 4 trimethylation (K4), histone 3 lysine 27 trimethylation (K27), both marks (K4 + K27) or neither mark. Click here for file

Additional file 4 **Figure S2**. No correlation between body weight and open field activity. The body weights of 10 *Trim28^+/+ ^*mice and 14 *Trim28^MommeD9/+ ^*mice were plotted against their activity in an open field test (Squares). Click here for file
