# Supplementary material for: Reduced levels of two modifiers of epigenetic gene silencing, Dnmt3a and Trim28, cause increased phenotypic noise
Source: Genome Biol. 2010 Nov 19;11(11):R111. doi: 10.1186/gb-2010-11-11-r111 (PMC3156950; doi:10.1186/gb-2010-11-11-r111)
Supplement: Additional file 1 — Table S1. List of genes in the MommeD9 linked interval. [file gb-2010-11-11-r111-S1.docx]

| **Gene Start (bp)** | **Ensembl Gene ID** | **Gene Name** | **Description** |
| --- | --- | --- | --- |
| 13551213 | ENSMUSG00000004500 | Zfp324 | zinc finger protein 324 |
| 13563197 | ENSMUSG00000033961 | Zfp446 | zinc finger protein 446 |
| 13573701 | ENSMUSG00000030382 | Slc27a5 | solute carrier family 27 (fatty acid transporter), member 5 |
| 13591060 | ENSMUSG00000049600 | Zbtb45 | zinc finger and BTB domain containing 45 |
| 13609758 | ENSMUSG00000005566 | Trim28 | tripartite motif-containing 28 |
| 13617368 | ENSMUSG00000033916 | Chmp2a | chromatin modifying protein 2A |
| 13620588 | ENSMUSG00000005575 | Ube2m | ubiquitin-conjugating enzyme E2M (UBC12 homolog, yeast) |
| 13627710 | ENSMUSG00000030380 | Mzf1 | myeloid zinc finger 1 |
| 13669638 | ENSMUSG00000070817 | V1rj3 | vomeronasal 1 receptor, J3 |
| 13687472 | ENSMUSG00000070816 | AC121301.8 | Vomeronasal receptor 1 E8 (Vomeronasal receptor V1RJ1) |
| 13716820 | ENSMUSG00000070815 | V1rk1 | vomeronasal 1 receptor, K1 |
| 13804688 | ENSMUSG00000050453 | V1rj2 | vomeronasal 1 receptor, J2 |
| 13844316 | ENSMUSG00000070814 | 6330408A02Rik | RIKEN cDNA 6330408A02 gene |
| 13862632 | ENSMUSG00000056394 | Lig1 | ligase I, DNA, ATP-dependent |
| 13911089 | ENSMUSG00000033847 | Pla2g4c | phospholipase A2, group IVC (cytosolic, calcium-independent) |
| 13983481 | ENSMUSG00000005649 | Cabp5 | calcium binding protein 5 |
| 14036190 | ENSMUSG00000074378 | Gm767 | gene model 767, (NCBI) |
| 14126739 | ENSMUSG00000078800 | 9230107M04Rik | RIKEN cDNA 9230107M04 gene |
| 14318908 | ENSMUSG00000070811 | C730007P19Rik | RIKEN cDNA C730007P19 gene |
| 14381602 | ENSMUSG00000078798 | Sult2a1 | sulfotransferase family 2A, dehydroepiandrosterone-preferring 2 |
| 14495026 | ENSMUSG00000074377 | AC123600.1 | sulfotransferase family member-like |
| 14807730 | ENSMUSG00000070810 | EG629219 | predicted gene, EG629219 |
| 14996068 | ENSMUSG00000030378 | 2810007J24Rik | RIKEN cDNA 2810007J24 gene |
| 15146518 | ENSMUSG00000041596 | B430211C08Rik | RIKEN cDNA B430211C08 gene |
| 15245057 | ENSMUSG00000055942 | EG194588 | predicted gene, EG194588 |
| 15364018 | ENSMUSG00000064977 | U6 | U6 spliceosomal RNA |
| 15497707 | ENSMUSG00000075887 | U6 | U6 spliceosomal RNA |
| 15982194 | ENSMUSG00000074372 | AC165150.3-203 | OBOX2 |
| 16065174 | ENSMUSG00000066772 | Obox3 | oocyte specific homeobox 3 |
| 16132609 | ENSMUSG00000054310 | Obox1 | oocyte specific homeobox 1 |
| 16178580 | ENSMUSG00000075966 | U6 | U6 spliceosomal RNA |
| 16280562 | ENSMUSG00000075896 | U6 | U6 spliceosomal RNA |
| 16335719 | ENSMUSG00000074366 | Obox5 | oocyte specific homeobox 5 |
| 16418599 | ENSMUSG00000041583 | Obox6 | oocyte specific homeobox 6 |
| 16451298 | ENSMUSG00000041578 | Crx | cone-rod homeobox containing gene |
| 16467966 | ENSMUSG00000074365 | Crxos1 | Crx opposite strand transcript 1 |
| 16502559 | ENSMUSG00000041571 | Sepw1 | selenoprotein W, muscle 1 |
| 16523187 | ENSMUSG00000041560 | Gltscr2 | glioma tumor suppressor candidate region gene 2 |
| 16524111 | ENSMUSG00000080478 | SNORD23 | small nucleolar RNA, C/D box 23 |
| 16532328 | ENSMUSG00000074364 | Ehd2 | EH-domain containing 3 |
| 16556611 | ENSMUSG00000070808 | Gltscr1 | glioma tumor suppressor candidate region gene 1 |
| 16657291 | ENSMUSG00000078796 | Zfp541 | zinc finger protein 541 |
| 16683984 | ENSMUSG00000006024 | Napa | N-ethylmaleimide sensitive fusion protein attachment protein alpha |
| 16705244 | ENSMUSG00000006021 | Kptn | kaptin |
| 16715509 | ENSMUSG00000030376 | Slc8a2 | solute carrier family 8 (sodium/calcium exchanger), member 2 |
| 16760748 | ENSMUSG00000041420 | Meis3 | Meis homeobox 3 |
| 16782496 | ENSMUSG00000006019 | Dhx34 | DEAH (Asp-Glu-Ala-His) box polypeptide 34 |
| 16820087 | ENSMUSG00000080413 | U6atac | Novel snRNA |
| 16820101 | ENSMUSG00000074361 | Gpr77 | G protein-coupled receptor 77 |
| 16832092 | ENSMUSG00000049130 | C5ar1 | complement component 5a receptor 1 |
| 16859394 | ENSMUSG00000041375 | Ccdc9 | coiled-coil domain containing 9 |
| 16895002 | ENSMUSG00000002083 | Bbc3 | BCL2 binding component 3 |

Data is based on the NCBI m37 mouse assembly
