# Supplementary material for: Reduced levels of two modifiers of epigenetic gene silencing, Dnmt3a and Trim28, cause increased phenotypic noise
Source: Genome Biol. 2010 Nov 19;11(11):R111. doi: 10.1186/gb-2010-11-11-r111 (PMC3156950; doi:10.1186/gb-2010-11-11-r111)
Supplement: Additional file 2 — Table S2. Gene expression analysis of Trim28MommeD9/+ mice. [file gb-2010-11-11-r111-S2.docx]

| **GENE** | **FOLD CHANGE** | **DIFFERENCE SCORE** |
| --- | --- | --- |
| Mas1 | 7.83 | -46.68 |
| 1700037H04Rik | 1.95 | -31.95 |
| Lage3 | 1.21 | -23.99 |
| Dok2 | 1.58 | -23.40 |
| S100a1 | 1.31 | -23.30 |
| Gm2a | 1.39 | -23.30 |
| Gstm6 | 1.82 | -21.03 |
| Dcxr | 1.25 | -20.12 |
| Fmr1nb | 9.92 | -19.18 |
| Spsb2 | 1.27 | -18.97 |
| 1810049H13Rik | 1.28 | -18.97 |
| Pxmp2 | 1.35 | -18.97 |
| Gstt2 | 1.52 | -18.96 |
| MGC18837 | 1.16 | -18.67 |
| Vwf | 1.38 | -18.67 |
| Gstt1 | 1.29 | -18.51 |
| Ocln | 1.67 | -18.48 |
| Ctsh | 1.20 | -18.23 |
| Hibadh | 1.09 | -18.21 |
| Serping1 | 1.11 | -18.16 |
| Bcat2 | 1.50 | -17.95 |
| Blvra | 1.20 | -17.95 |
| Gstk1 | 1.14 | -17.92 |
| Gsta4 | 1.34 | -17.90 |
| Gdpd3 | 1.67 | -17.90 |
| A430084P05Rik | 1.50 | -17.85 |
| Ephx1 | 1.23 | -17.82 |
| Sdc3 | 1.36 | -17.67 |
| BC006662 | 1.20 | -17.50 |
| Anks1 | 1.34 | -17.46 |
| Gamt | 1.41 | -17.41 |
| Slc2a1 | 1.29 | -17.39 |
| Fcna | 1.42 | -17.29 |
| Ddah1 | 1.27 | -17.24 |
| Evc2 | 1.57 | -17.14 |
| Prodh2 | 1.26 | -17.09 |
| Cotl1 | 1.15 | -17.09 |
| EG433923 | 1.14 | -17.03 |
| LOC100046650 | 1.25 | -16.94 |
| Gsto1 | 1.20 | -16.88 |
| Pcgf1 | 1.33 | -16.87 |
| C4b | 1.17 | -16.68 |
| Ftcd | 1.25 | -16.65 |
| Pea15 | 1.20 | -16.54 |
| Slc25a10 | 1.32 | -16.50 |
| Rxrg | 1.35 | -16.48 |
| Myh10 | 1.78 | -16.47 |
| Cyp2a12 | 1.14 | -16.47 |
| C2 | 1.24 | -16.36 |
| Slc6a13 | 1.15 | -16.29 |
| Hcst | 1.42 | -16.25 |
| Col4a2 | 1.25 | -16.23 |
| Pex11b | 1.23 | -16.21 |
| 5033414D02Rik | 1.43 | -16.19 |
| Dscr2 | 1.15 | -16.10 |
| Bola3 | 1.20 | -16.10 |
| Vsig4 | 1.34 | -16.10 |
| Cyp4f14 | 1.18 | -16.09 |
| P2ry13 | 1.40 | -16.09 |
| Acin1 | -1.89 | 16.01 |
| Tor2a | -2.08 | 16.03 |
| Wdr23 | -2.42 | 16.09 |
| Rgs10 | -1.81 | 16.09 |
| Dcun1d3 | -1.77 | 16.09 |
| Actr3 | -1.67 | 16.09 |
| Abcb11 | -1.31 | 16.09 |
| Mrpl52 | -2.06 | 16.09 |
| Mbtd1 | -1.70 | 16.09 |
| Cdc42ep2 | -1.20 | 16.09 |
| H2-Q10 | -1.17 | 16.09 |
| Hyou1 | -1.65 | 16.10 |
| Ctsb | -1.50 | 16.10 |
| Nudt4 | -1.42 | 16.10 |
| L3mbtl2 | -1.63 | 16.10 |
| Rrn3 | -3.02 | 16.11 |
| Stk24 | -1.32 | 16.12 |
| 2610016F04Rik | -1.85 | 16.15 |
| Nt5c | -1.67 | 16.15 |
| Vdac2 | -1.46 | 16.17 |
| Acsm5 | -1.34 | 16.18 |
| Ddx3x | -4.45 | 16.19 |
| Nudcd2 | -3.68 | 16.19 |
| Rgn | -3.49 | 16.19 |
| Dld | -3.45 | 16.19 |
| Bcas2 | -2.96 | 16.19 |
| 1300007F04Rik | -2.33 | 16.19 |
| Npc2 | -2.26 | 16.19 |
| Vps36 | -1.35 | 16.19 |
| Adipor2 | -1.26 | 16.19 |
| Ociad1 | -1.22 | 16.21 |
| Afm | -2.21 | 16.23 |
| Hisppd1 | -1.95 | 16.23 |
| Cct8 | -1.57 | 16.23 |
| Exosc8 | -1.83 | 16.25 |
| D15Ertd621e | -1.71 | 16.25 |
| Zfp612 | -1.53 | 16.25 |
| Arl1 | -1.44 | 16.25 |
| Abca8a | -1.37 | 16.25 |
| LOC100046775 | -1.34 | 16.25 |
| Gps2 | -1.18 | 16.25 |
| Spast | -1.78 | 16.29 |
| Alas1 | -3.09 | 16.31 |
| Arf6 | -1.44 | 16.31 |
| C730025P13Rik | -2.67 | 16.33 |
| Psat1 | -1.65 | 16.33 |
| Spp1 | -2.31 | 16.36 |
| Kmo | -2.25 | 16.37 |
| Pex14 | -1.30 | 16.38 |
| S100a10 | -2.02 | 16.40 |
| Gkap1 | -1.40 | 16.42 |
| Rbbp9 | -2.34 | 16.43 |
| Snap29 | -1.63 | 16.43 |
| Angptl3 | -2.17 | 16.44 |
| LOC100047935 | -1.43 | 16.47 |
| Otud5 | -1.54 | 16.47 |
| Tcp1 | -1.56 | 16.49 |
| Thumpd1 | -1.60 | 16.59 |
| Mlycd | -1.54 | 16.59 |
| Triap1 | -1.12 | 16.59 |
| Ptges3 | -2.96 | 16.61 |
| Eif3k | -1.94 | 16.61 |
| Atpbd1b | -1.87 | 16.61 |
| Slc30a1 | -1.71 | 16.61 |
| LOC100042777 | -1.57 | 16.61 |
| Kynu | -1.43 | 16.61 |
| Coro1c | -1.31 | 16.61 |
| Tpp2 | -1.79 | 16.61 |
| Jak1 | -1.33 | 16.61 |
| Slc37a4 | -2.32 | 16.63 |
| Slc30a6 | -1.97 | 16.63 |
| Med28 | -1.80 | 16.63 |
| LOC100043391 | -1.34 | 16.63 |
| EG433182 | -3.04 | 16.65 |
| Ccnl1 | -2.70 | 16.65 |
| LOC547343 | -2.37 | 16.65 |
| Rev3l | -2.20 | 16.65 |
| Dcun1d5 | -1.88 | 16.65 |
| LOC545056 | -1.79 | 16.65 |
| Zfp91-cntf | -1.51 | 16.65 |
| Ywhag | -1.39 | 16.65 |
| 4833418A01Rik | -1.33 | 16.65 |
| Gja1 | -1.20 | 16.65 |
| Sec63 | -1.23 | 16.66 |
| Arl8a | -2.40 | 16.67 |
| 1110002B05Rik | -2.38 | 16.67 |
| Chchd7 | -2.26 | 16.67 |
| Lman1 | -1.45 | 16.67 |
| 2610002M06Rik | -1.92 | 16.70 |
| Trip12 | -1.57 | 16.71 |
| Ascc3l1 | -1.28 | 16.73 |
| Nhp2l1 | -1.40 | 16.73 |
| Tfdp2 | -1.50 | 16.76 |
| LOC668492 | -1.26 | 16.76 |
| Cnih4 | -1.22 | 16.77 |
| Nfkbid | -1.43 | 16.79 |
| Lyar | -2.84 | 16.85 |
| Cdc42ep3 | -2.22 | 16.85 |
| Larp2 | -2.11 | 16.85 |
| Sh3bgrl | -1.89 | 16.85 |
| Coq2 | -1.76 | 16.85 |
| Rpo1-1 | -1.94 | 16.87 |
| Mars | -1.21 | 16.88 |
| Polr2g | -2.33 | 16.90 |
| Ugt2b34 | -3.51 | 16.92 |
| LOC100045882 | -1.20 | 16.92 |
| Zc3h15 | -2.78 | 16.94 |
| Rnasek | -1.55 | 17.00 |
| Epb4.1 | -1.63 | 17.02 |
| Sec16b | -1.39 | 17.03 |
| Eed | -3.30 | 17.05 |
| Manbal | -1.26 | 17.06 |
| Ndufb9 | -3.04 | 17.09 |
| Arih1 | -2.07 | 17.09 |
| Alcam | -1.63 | 17.09 |
| Znrf1 | -1.62 | 17.09 |
| EG625054 | -1.32 | 17.09 |
| 4933424B01Rik | -1.58 | 17.09 |
| Ugt2b35 | -1.45 | 17.14 |
| Cul1 | -1.89 | 17.15 |
| Rad23b | -1.29 | 17.15 |
| Psmc1 | -1.22 | 17.15 |
| 6330577E15Rik | -3.08 | 17.16 |
| Slc10a1 | -1.90 | 17.16 |
| Atp5h | -1.33 | 17.16 |
| Tcof1 | -2.15 | 17.20 |
| 2900064A13Rik | -3.77 | 17.21 |
| D10Wsu52e | -1.35 | 17.29 |
| Acsl1 | -1.26 | 17.30 |
| Mrps15 | -2.34 | 17.32 |
| Tmem19 | -2.19 | 17.37 |
| 2210412D01Rik | -2.97 | 17.38 |
| Purb | -1.37 | 17.40 |
| Ankrd46 | -2.49 | 17.41 |
| Cog2 | -1.42 | 17.41 |
| Dpyd | -2.70 | 17.46 |
| Hsdl1 | -2.43 | 17.46 |
| Mfap1b | -2.22 | 17.46 |
| Irak1 | -2.19 | 17.46 |
| Aldh1a7 | -1.70 | 17.46 |
| Lyrm1 | -1.64 | 17.46 |
| LOC216443 | -1.19 | 17.46 |
| Dhx33 | -1.80 | 17.50 |
| Rbm8a | -2.15 | 17.52 |
| Myo1b | -1.75 | 17.52 |
| Ccdc5 | -1.38 | 17.52 |
| Mapbpip | -1.71 | 17.54 |
| LOC435565 | -2.93 | 17.56 |
| Dtd1 | -1.36 | 17.60 |
| AK129128 | -1.36 | 17.61 |
| Kctd2 | -1.75 | 17.63 |
| Dhx36 | -1.29 | 17.63 |
| LOC100047794 | -3.34 | 17.67 |
| BC085271 | -1.84 | 17.67 |
| Sec14l2 | -1.71 | 17.67 |
| Plk2 | -1.55 | 17.67 |
| Wtip | -1.21 | 17.67 |
| Pom121 | -1.20 | 17.67 |
| Serpina1e | -2.13 | 17.76 |
| Man1a | -1.83 | 17.76 |
| Mrpl20 | -1.95 | 17.82 |
| 2010011I20Rik | -1.32 | 17.82 |
| Ccdc25 | -2.31 | 17.85 |
| Uhrf1bp1l | -1.63 | 17.85 |
| 0610038F07Rik | -1.57 | 17.85 |
| Lpgat1 | -2.62 | 17.92 |
| Zfp825 | -1.83 | 17.99 |
| Mtmr1 | -2.18 | 18.03 |
| Wnt2 | -2.28 | 18.06 |
| Elovl2 | -2.41 | 18.16 |
| Dmgdh | -2.00 | 18.16 |
| BC005537 | -1.92 | 18.16 |
| Nudt21 | -1.75 | 18.16 |
| Psmd8 | -1.67 | 18.16 |
| Uqcrc2 | -1.29 | 18.16 |
| Serpina9 | -1.63 | 18.21 |
| LOC100046320 | -2.27 | 18.23 |
| BC020002 | -1.78 | 18.23 |
| Bad | -1.15 | 18.23 |
| Actl6a | -2.52 | 18.27 |
| March6 | -1.27 | 18.29 |
| Snx10 | -1.48 | 18.35 |
| EG277333 | -2.26 | 18.45 |
| Bace1 | -1.60 | 18.45 |
| Ylpm1 | -1.60 | 18.45 |
| D14Ertd449e | -1.49 | 18.45 |
| Nmd3 | -2.47 | 18.51 |
| Ergic2 | -1.98 | 18.60 |
| Crlf3 | -1.53 | 18.61 |
| Eif3s8 | -1.30 | 18.64 |
| Aco1 | -1.71 | 18.65 |
| Dera | -1.66 | 18.65 |
| 9530008L14Rik | -1.46 | 18.65 |
| Eif2s3x | -1.24 | 18.65 |
| Alb | -3.59 | 18.67 |
| Arl5a | -2.11 | 18.67 |
| Pa2g4 | -1.96 | 18.67 |
| Bzw1 | -1.80 | 18.67 |
| Cdc16 | -1.74 | 18.67 |
| 5730469M10Rik | -1.43 | 18.67 |
| 0610010K06Rik | -1.24 | 18.67 |
| Tor1b | -1.82 | 18.70 |
| Prpf6 | -1.78 | 18.70 |
| LOC100046891 | -1.27 | 18.71 |
| 4432416J03Rik | -2.10 | 18.84 |
| Ubfd1 | -2.54 | 18.88 |
| Hnrpab | -1.36 | 18.89 |
| Akr1e1 | -2.39 | 18.90 |
| 1190002N15Rik | -2.19 | 18.90 |
| Tmem32 | -1.59 | 18.97 |
| Cggbp1 | -1.48 | 18.97 |
| Nxf1 | -1.46 | 18.97 |
| Asb8 | -1.20 | 18.97 |
| Otud6b | -1.88 | 18.99 |
| Zc3h18 | -1.59 | 19.11 |
| Atp5e | -2.54 | 19.20 |
| D5Ertd579e | -1.64 | 19.20 |
| Gnb2l1 | -1.98 | 19.59 |
| Rassf3 | -6.51 | 20.00 |
| 2610304G08Rik | -1.92 | 22.26 |
| Amy2 | -2.89 | 23.30 |
| Poli | -1.39 | 23.30 |
| Sp5 | -1.86 | 23.40 |
| Cbr1 | -1.64 | 23.99 |
| Nckap1 | -1.31 | 23.99 |
