# Supplementary figures and images for: Reduced levels of two modifiers of epigenetic gene silencing, Dnmt3a and Trim28, cause increased phenotypic noise
Source: Genome Biol. 2010 Nov 19;11(11):R111. doi: 10.1186/gb-2010-11-11-r111 (PMC3156950; doi:10.1186/gb-2010-11-11-r111)

**A**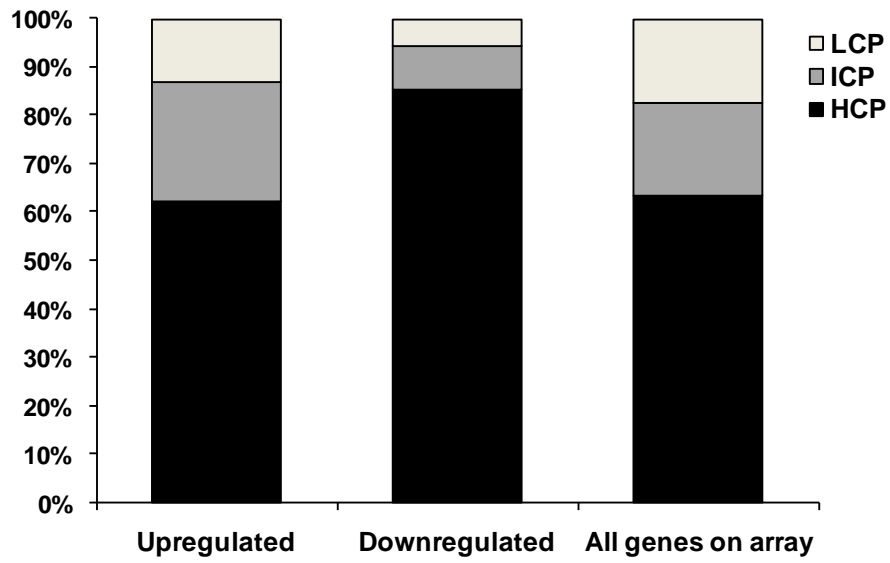**B**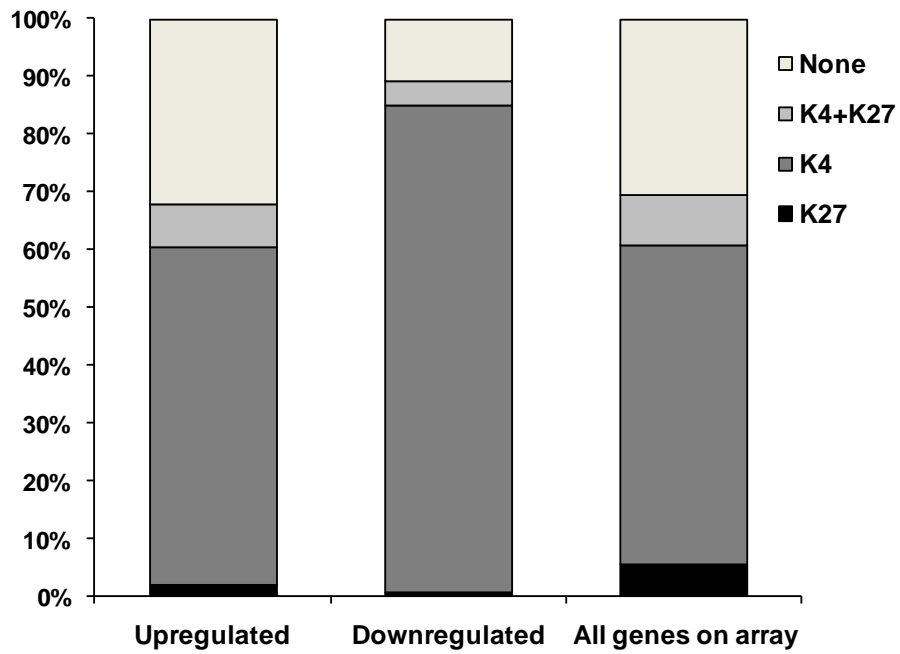

Supplement: Additional file 3 — Figure S1. Promoter characteristics of aberrantly expressed genes in Trim28MommeD9/+ mice. Genome-wide expression analysis (Illumina MouseRef-8 v2.0 Expression BeadChip) was performed using RNA from the livers of 4-week-old male Trim28MommeD9/+ individuals (n = 4) and their wild-type littermates (n = 4). Promoter classification analysis was performed on genes classed as upregulated (n = 59) and downregulated (n = 225) by the GenomeStudio Gene Expression Module (Illumina). (a) Promoters were classified as low (LCP), intermediate (ICP) or high (HCP) CpG density. (b) Promoters were classified as having histone 3 lysine 4 trimethylation (K4), histone 3 lysine 27 trimethylation (K27), both marks (K4 + K27) or neither mark. [file gb-2010-11-11-r111-S3.pdf]

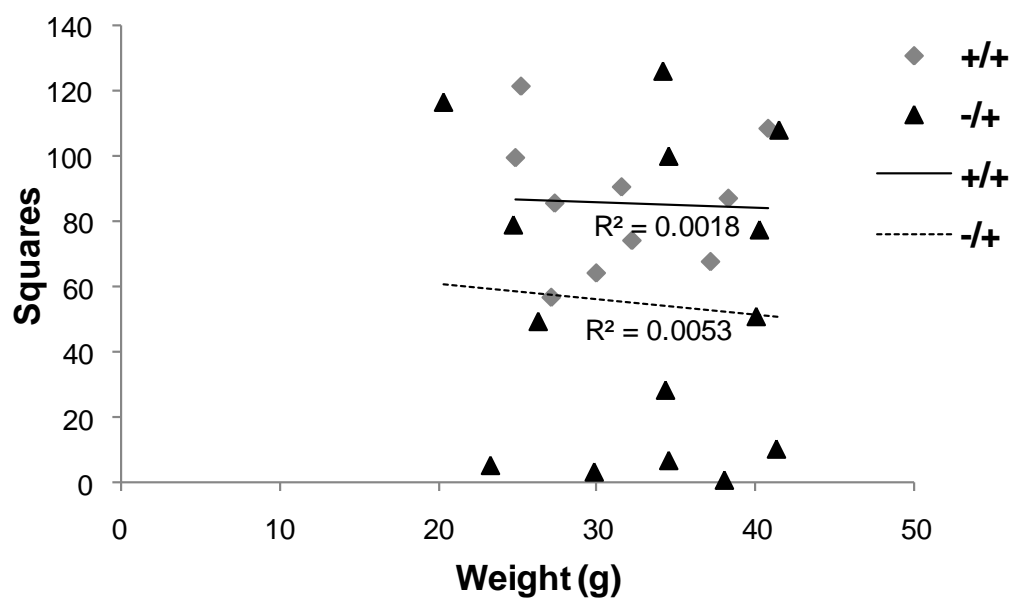

Supplement: Additional file 4 — Figure S2. No correlation between body weight and open field activity. The body weights of 10 Trim28+/+ mice and 14 Trim28MommeD9/+ mice were plotted against their activity in an open field test (Squares). [file gb-2010-11-11-r111-S4.pdf]
